# IRES-Containing VEEV Vaccine Protects Cynomolgus Macaques from IE Venezuelan Equine Encephalitis Virus Aerosol Challenge

**DOI:** 10.1371/journal.pntd.0003797

**Published:** 2015-05-28

**Authors:** Shannan L. Rossi, Kasi E. Russell-Lodrigue, Stephanie Z. Killeen, Eryu Wang, Grace Leal, Nicholas A. Bergren, Heather Vinet-Oliphant, Scott C. Weaver, Chad J. Roy

**Affiliations:** 1 Institute of Human Infection and Immunity, Sealy Center for Vaccine Development and Department of Pathology, University of Texas Medical Branch, Galveston, Texas, United States of America; 2 Divisions of Veterinary Medicine and Microbiology, Tulane National Primate Research Center, Covington, Louisiana, United States of America; 3 Department of Microbiology and Immunology, University of Texas Medical Branch, Galveston, Texas, United States of America; 4 Department of Microbiology and Immunology, Tulane School of Medicine, New Orleans, Louisiana, United States of America; Centers for Disease Control and Prevention, UNITED STATES

## Abstract

Venezuelan equine encephalitis virus (VEEV) is an arbovirus endemic to the Americas that is responsible for severe, sometimes fatal, disease in humans and horses. We previously described an IRES-based VEE vaccine candidate based up the IE serotype that offers complete protection against a lethal subtype IE VEEV challenge in mice. Here we demonstrate the IRES-based vaccine’s ability to protect against febrile disease in cynomolgus macaques. Vaccination was well tolerated and elicited robust neutralizing antibody titers noticed as early as day 14. Moreover, complete protection from disease characterized by absence of viremia and characteristic fever following aerosolized IE VEEV challenge was observed in all vaccinees compared to control animals, which developed clinical disease. Together, these results highlight the safety and efficacy of IRES-based VEEV vaccine to protect against an endemic, pathogenic VEEV IE serotype.

## Author Summary

Venezuelan equine encephalitis virus (VEEV) is a mosquito-borne arbovirus endemic to the Americas that affects a wide range of equids and humans. Vaccination has been one of the strategies to combat spread of disease in areas with high rates incidence of VEEV, although existing vaccines have proven less than effective against genetically diverse serotypes. In addition to being a natural vectorborne threat, VEEV is considered a biological threat agent that could be used as a weapon. We evaluated a new Internal Ribosome Entry Site (IRES)-containing chimeric viral vaccine using an advanced nonhuman primate model of VEEV infection. Vaccinated animals showed robust humoral immune responses to a single prime immunization with IE VEEV/IRES vaccine. The vaccine protected against an aerosolized IE (68U201) challenge, with vaccinees showing no blood viremia or development of febrile disease, including no pyrexia associated with VEEV infection. This vaccine product has shown efficacy against serotype-specific challenge model and provides enabling data as the basis for future clinical development.

## Introduction

Venezuelan equine encephalitis (VEE) virus (VEEV), an arbovirus with a wide geographic distribution across North, Central and South America, causes periodic outbreaks in human and equine populations as well as endemic disease following spillover from enzootic transmission cycles [1]. There are several subtypes of VEEV based upon antigenic profiles. The epizootic/epidemic strains associated with equine-amplified outbreaks, IAB and IC, arise from constantly circulating progenitor enzootic ID strains. The endemic ID and IE subtypes are often overlooked as causes of human disease due to their overlap in signs and symptoms with dengue and other acute febrile tropical diseases. However, outbreaks of subtype IE VEEV in Mexico during the 1990s involving fatal infections of horses showed that this enzootic subtype can cause overt disease in both humans and horses [2]. Surveillance for acute febrile illness has demonstrated that enzootic subtype ID and IE VEEV strains can produce symptomologies similar to those caused by the more virulent epizootic/epidemic strains [3]. A recent study showed that subtype IE VEEV continuously circulates in the Gulf Coast region of Mexico as evidenced by a high percentage of seropositive horses, cattle and humans [4]. However, as in many parts of Latin America where laboratory diagnostics are not widely available, human infections are typically misdiagnosed as dengue [1]. These data highlight the need for continued surveillance and control strategies for VEEV in this region.

Several documented human VEEV infections have occurred in the laboratory setting from punctures (e.g., needle sticks) and inhalation of inadvertently generated aerosolized particles. In addition to being highly infectious by the aerosol route, VEEV replicates to high titers *in vitro* and is relatively stable in otherwise normal environmental conditions. It is because of these characteristics, paired with a legacy of offensive bioweapon development that makes VEEV a biological threat agent and a select agent.

Vaccines provide the best means of preventing VEE. The only vaccine approved for investigational new drug use in humans, TC-83, was developed in 1961. The mechanism of attenuation is based on one missense mutation in the E2 envelope glycoprotein gene and one nucleotide substitution in the 5’ untranslated genome region [5]. The reliance on only two point mutations is believed to explain TC-83’s reactogenicity and potential for reversion to a wild-type-like pathogenic phenotype. Furthermore, there is limited cross-neutralization between IAB and IE subtype viruses [6], and the TC-83 vaccine provides limited protection against subtype IE [7].

Nonhuman primates (NHP) have been used as a model for human VEE, and can be readily infected via needle inoculation or aerosol inhalation. Common features to peripheral routes VEEV infection include an abrupt onset of fever, viremia and lymphopenia lasting a few days [8]. NHPs infected with VEEV by the aerosol route also present with signs similar to human VEE, including an acute viremia lasting several days accompanied by fever but rarely resulting in neurological disease [9]. A model of adult cynomolgus macaques (*Macaca fascicularis*) infected with subtype IE strain 68U201 by the aerosol route was previously described [10]. This model was able to reliably reproduce viremia, fever, and clinical illness in the animals.

We recently described the development and characterization of a VEE vaccine candidate based upon the insertion of an Internal Ribosome Entry Site (IRES) upstream of the structural polyprotein open reading frame [11]. This IE VEEV/IRESv1 vaccine was attenuated *in vitro* as well as being safe and efficacious in the mouse model. Vaccinated mice produced neutralizing antibodies by three weeks post infection and were fully protected against lethal 68U201 VEEV infection at the earliest (1 month post vaccination) or latest (1 year post infection) challenge times tested. To further test this vaccine in the cynomolgus macaque model, we immunized animals with a single dose, then challenged by aerosol with virulent VEEV to assess protection from development of clinical disease.

## Materials and Methods

### Cell lines

Vero (African green monkey kidney) cells were grown in Dulbecco’s minimum essential media (DMEM) supplemented with 5% fetal bovine serum (FBS) and 1% penicillin/streptomycin (P/S). Cells were stored at 37°C in 5% CO_2_.

### Animals

Age- and sex-matched cynomolgus macaques (*Macaca fascicularis*) weighing 3–6 kg, free of simian immunodeficiency virus (SIV), simian type D retrovirus, simian T-lymphotropic virus, and antibodies against western, Venezuelan, and eastern equine encephalitis, Sindbis, Semliki Forest, and chikungunya alphaviruses (assayed by hemagglutination inhibition) were used.

### Ethics statement

All experiments using cynomolgus macaques were approved by the Tulane Institutional Animal Care and Use Committee (Protocol P0171). The Tulane National Primate Research Center (TNPRC) is an Association for Assessment and Accreditation of Laboratory Animal Care International accredited facility (AAALAC#000594). The U.S. National Institutes of Health (NIH) Office of Laboratory Animal Welfare assurance number for the TNPRC is A3071-01. Nonhuman primate housing consisted of individual open metal caging units that allowed visual recognition and protected contact with other study animals in the room. Animals were maintained on standard primate chow supplemented daily with fresh fruits and vegetables. Animals were provided standard environmental enrichment during this study, which included manipulable items in cage, perches, foraging/task-oriented feeding methods, and human interactions with caretakers and research staff. All clinical procedures, including administration of anesthesia and analgesics, were carried out under the direction of a laboratory animal veterinarian. Animals were anesthetized with ketamine hydrochloride for blood collection procedures. Animals were pre-anesthetized with ketamine hydrochloride, acepromazine, and glycopyrolate, intubated and maintained on a mixture of isoflurane and oxygen for telemetric implantation surgeries. Buprenorphine was given intra-operatively and post-operatively for analgesia. All possible measures are taken to minimize discomfort of all the animals used in this study. Animals were closely monitored daily following surgery for any signs of illness such as anorexia, lethargy, diarrhea, vomiting, and dehydration. Appropriate medical care was implemented if any of these signs of illness were noted. If euthanasia was required in the judgment of the TNPRC veterinary staff, animals were euthanized in accordance with the recommendations of the panel on Euthanasia of the American Veterinary Medical Association. The standard method of euthanasia for nonhuman primates at the TNPRC is anesthesia with ketamine hydrochloride (10 mg/kg) followed by an overdose of sodium pentobarbital. Tulane University complies with NIH policy on animal welfare, the Animal Welfare Act, and all other applicable federal, state and local laws.

Subcutaneous radio telemetry transmitters combined with sensors capable of detecting biopotential signals of an electrocardiogram (ECG) as well as thermistor type sensors capable of detecting temperature signals [T34G-8; Konigsberg Instruments (KI), Inc., Pasadena, CA] were surgically implanted under aseptic conditions. Following surgical implantation, animals were housed with cage-mounted antennas (TR38-1FG; Konigsberg Instruments, Inc.) configured to receive and transmit signals to a KI data acquisition base station. Data collection was continuous and recorded for periods of 48–72 hr using the CA Recorder [Data Integrated Scientific Systems (DISS), Dexter, MI]. Data parameters and analysis results recorded over the course of the study included mean analysis for body temperature measurements. Core body temperature was reported in hour averages for one-hour observation intervals, with each animal serving as their own control. Baseline pre-exposure data averages were generated from each respective control subject for a minimum of six days. Pre-exposure data were aligned by the time of day over a 24-hour period and averaged to establish detection thresholds. These thresholds of detection of significant events were defined to be greater than 1.5 times the maximum standard deviation of the pre-exposure control averages. Individual post exposure data were then aligned by time and compared against pre exposure values for each animal. An assessment of fever (hyperthermia) hours, hypothermia hours, and fever intensity was then performed for each animal. Significant changes among vaccination groups were compared statistically using the standard t-test.

### Virus manipulation and amplification

VEEV viruses were rescued after electroporating Vero cells with *in vitro*-transcribed viral RNA from the challenge strain 68U201 [10] or vaccine strain 68U201/IRESv1 [11] infectious clones. Supernatants from these cultures were harvested 24 hours later, clarified by centrifugation at 2000 rpm for 5 minutes to pellet cellular debris, then aliquoted and frozen at -80°C. The 68U201 stocks used for the aerosol challenge were passed one additional time in Vero cells to obtain a maximal titer. Approximately 48 hours after infection, supernatant from fifteen 68U201-infected flasks was pooled, and then clarified by centrifugation to remove cellular debris. Aliquots were frozen at -80°C then titrated in duplicate.

### Virus titration and cytopathic effect assays

All titrations were performed on confluent Vero cell monolayers as previously described [11]. Briefly, samples were diluted 10-fold in DMEM containing 2% FBS and 1% P/S, and a sample of each dilution was used to infect Vero cells. After 1 hour, an overlay containing the dilution media and 0.4% agarose was added. Cells were fixed with formaldehyde and strained with crystal violet following a 48-hour incubation. Titrations are expressed as plaque forming units (pfu)/ml.

Frozen tissue samples were thawed on ice, weighed, then 600 μl of DMEM supplemented with 2% FBS was added. Samples were homogenized in Qiagen Tissue Lyser II for 5 minutes at 26 p/sec, then spun for 3 minutes at 12k rpm to separate the media from the macerated tissue. A portion of the homogenate (50 μl) was placed onto a Vero cell monolayer and cytopathic effects were observed.

### Vaccination and aerosol exposures

Animals were randomly assigned to macaque cohorts; one (n = 5) was vaccinated subcutaneously (SC) with 68U201/IRESv1, and a second (n = 2) was sham-vaccinated SC with saline. Anesthetized animals were vaccinated SC in the upper deltoid with a single inoculation of either saline or 1x10^5^ pfu of vaccine in a volume of 100 μl. The animals were observed for signs of any clinically recognizable adverse responses visually and by remote radiotelemetry. Animals were bled on days 14, 21 and 49 following vaccination to assess antibody production.

On day 49 after vaccination, anesthetized macaques were challenged with VEEV IE strain 68U201 using a 16 liter head-only dynamic inhalation exposure system described previously [12], and monitored for 21 days.

### Gross and histopathological analyses

Necropsies were performed on animals when the study was terminated 21–23 days after challenge. Tissues were collected and frozen for viral titration as well as placed into 10% zinc-formalin for histopathological analysis: mandibular lymph node, pharyngeal lymph mode, tracheobronchial lymph node, mediastinal lymph node, lungs, spleen, liver, kidney, adrenal gland, thyroid, brain (five sites), and mesenteric lymph node. Frozen tissues were homogenized and measured for viral load by plaque assay on Vero cells, as were sera and cerebrospinal fluids (CSF).

### Plaque reduction neutralization assay

Plasma or sera were diluted 1/10 in PBS, then heat inactivated at 56°C for 1 hour prior to chilling to 4°C. Serial two-fold dilutions were performed in DMEM with 2% FBS. An equal volume of 68U201 VEEV virus diluted to 800 pfu/ml was added to each dilution. Following a one-hour incubation at 37°C, Vero monolayers were infected with 100ul of each dilution for another hour at 37°C. Monolayers were overlaid as described for plaque assay, then fixed at 48 hpi and plaques were visualized by crystal violet staining. PRNT_80_ titers were determined to be the dilution at which there was equal or more than an 80% reduction in plaques resulting from non-neutralization, respectively. All samples were assayed in duplicate, and the average titers were recorded.

## Results

To further evaluate the efficacy of the 68U201/IRESv1 vaccine, the superior NHP model for VEE disease was used. For these studies, seven cynomolgus macaques that were previously screened and found to be negative for prior alphavirus infections were used. Transponder chips to remotely measure vitals, including heart rate, respiratory rate and core body temperature, were implanted into each NHP prior to vaccination to ensure chip functionality and obtain baseline readings for each subject.

Each subject received a SC dose of 1x10^5^ pfu of 68U201/IRESv1 vaccine. None of the vaccinated animals showed detectable changes in physiological responses during the immunizations based upon the continuous telemetric monitoring. Sera analyzed from blood taken daily for three days following vaccination was absent of viremia (<10 pfu/ml).

**Fig 1:**
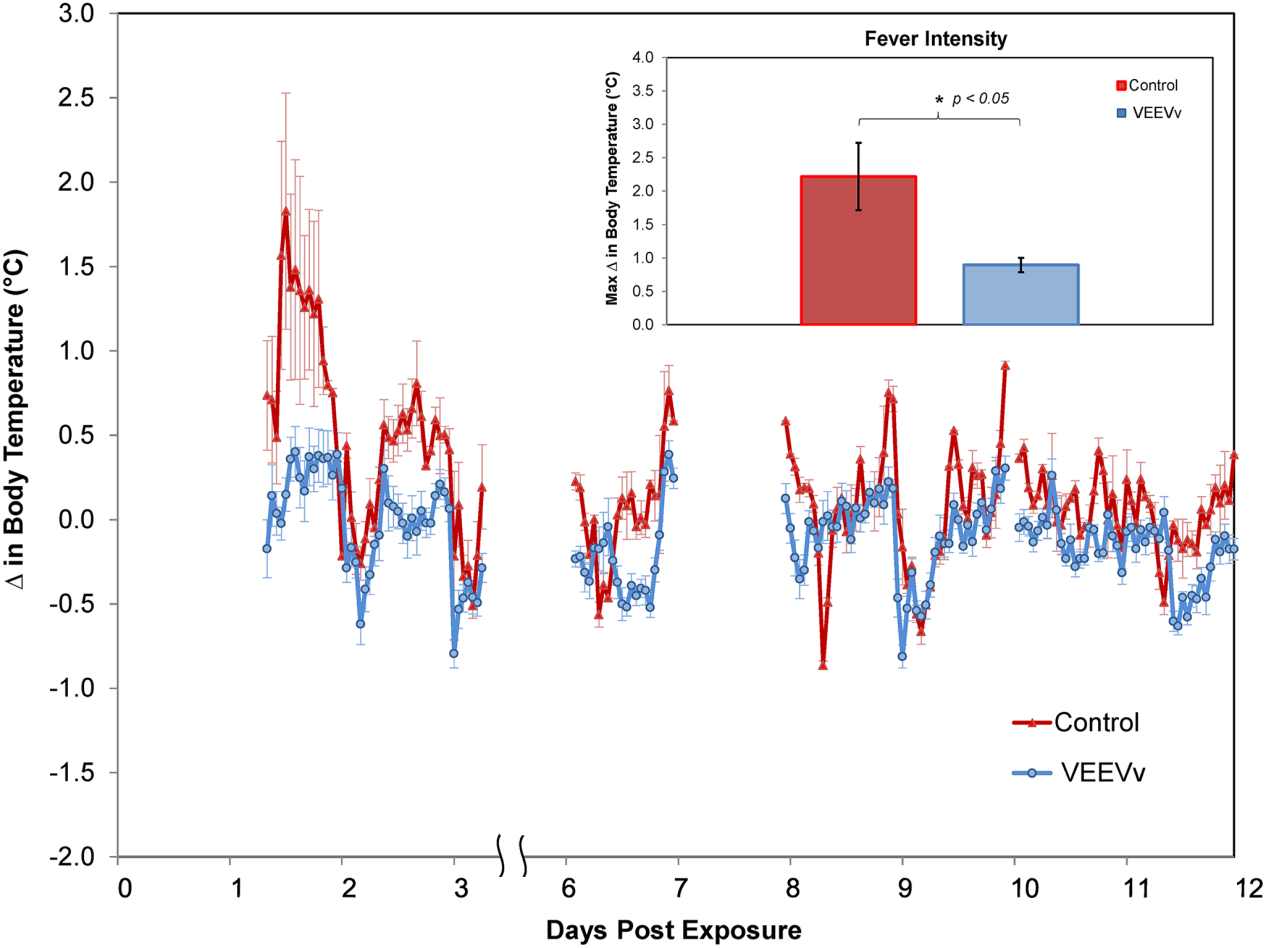
Core body temperature in vaccinated and unvaccinated primates after VEEV challenge. Physiological response of vaccinated cynomolgus macaques to VEEV challenge measured as changes in core body temperature for animals vaccinated with saline (red) or 68U201/IRESv1 (blue). Data were time-matched to pre-exposure values from each animal for derivation of relative changes. Significant changes in individual values were identified by divergence of more than 1.5 standard deviations from the pre-exposure value collected from the individual animal. Inset graph shows the difference in fever intensity among vaccination groups. Analysis (t-test) of vaccination group differences in fever intensity (mean ± SD) showed significant differences: saline (2.420 ± 1.143°C) and 68U201/IRESv1 (0.813 ± 0.348°C) with P = 0.012.

**Fig 2:**
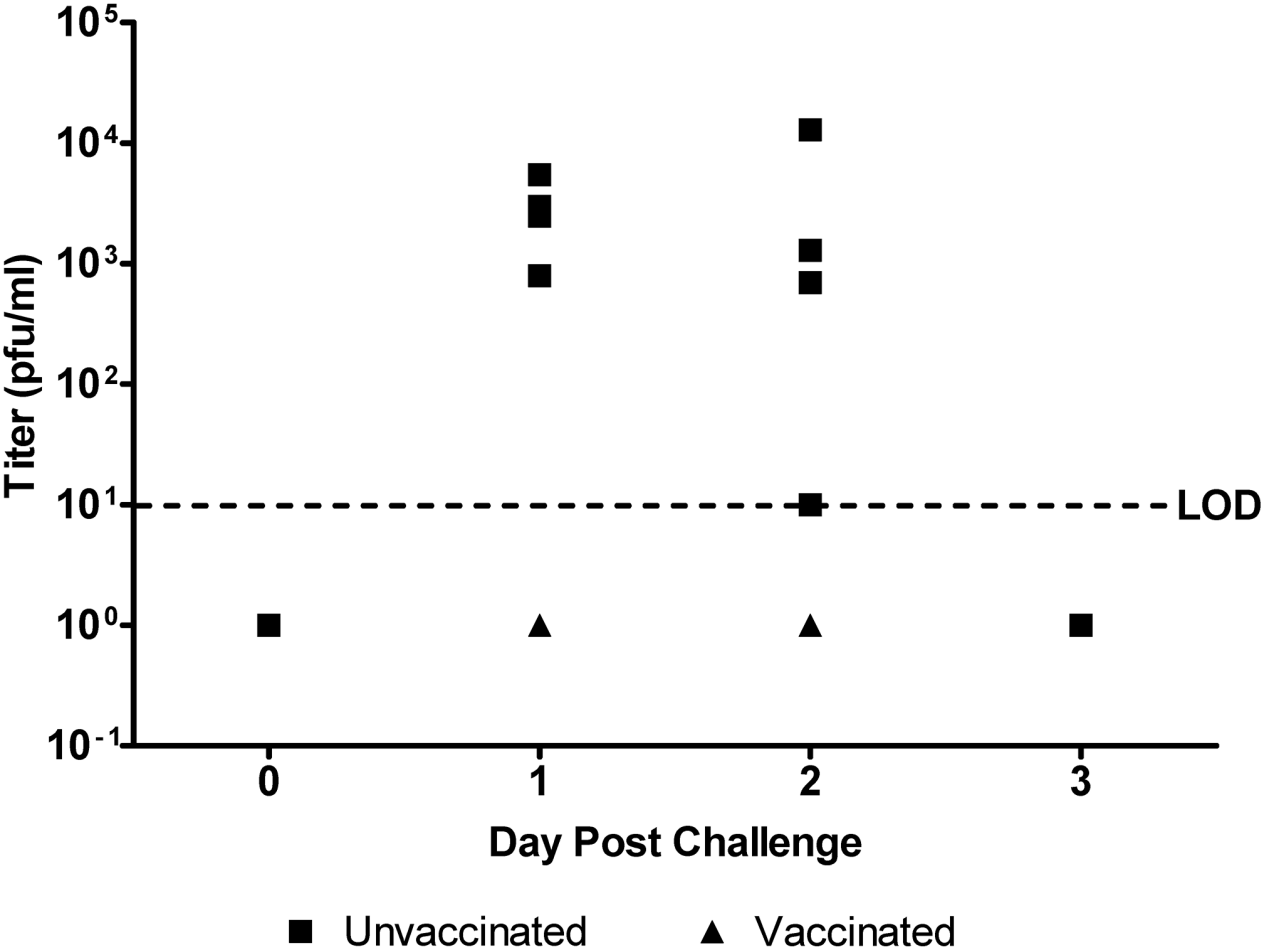
Viremia following aerosol exposure in vaccinated primates. Sera were titrated on Vero cells to determine levels of viremia for 3 days after 68U201 VEEV exposure. Individual points are shown for each vaccinated (triangle, n = 6) or unvaccinated (square, n = 4) NHP. The limit of detection was 10 pfu/ml. Serum samples with undetectable viremia are shown below this limit.

Aerosolized VEEV infection with the 68U201 strain, which is uniformly lethal in the mouse model, produces a nonlethal disease in NHP that is similar to that seen in humans [9]. Vaccine efficacy was therefore determined by protection from disease as measured by the absence of fever, a hallmark sign of clinical disease, and absence of viremia. All animals in the study were aerosol-challenged at an average inhaled dose of 4x10^4^ pfu/animal, approximately 0.5 log lower than our target dose of ≈1x10^5^ pfu/animal, based upon previous studies using similar modality of experimental challenge [10]. Slightly lower inhaled dose was due in part to unanticipated lower viral aerosol efficiencies observed using the IE strain used in the challenge. However, this challenge dose did produce measurable disease in the sham-vaccinated control animals, whereas vaccinees showed no observable changes in clinical or viremic status. These observations correlated with telemetry data that detected fever in the diseased control individuals lasting approximately 50 hours (Fig 1). Fever was characterized by elevated core body temperature as well as the loss of natural diurnal fluctuation in unprotected animals. Temperature changes were not observed in vaccinated animals, and these animals generally maintained normal diurnal temperature fluctuations postchallenge. Fever intensity, defined as the maximum deviation from baseline temperature for each particular animal within a group, was significantly higher (*p*<0.02) in sham-vaccinated animals compared to vaccinees (Fig 1, **inset graph**). It is worth noting that no data was collected between +80–140 and +170–190 hours postexposure due to a global loss of signal from the transponder acquisition hardware and thus data during this period of monitoring was unrecoverable. Subjects were bled daily for three days following aerosol exposure to quantify viremia (Fig 2). VEEV was only detected in the sera of unvaccinated NHPs for the first two days after challenge. All vaccinated NHP had undetectable viremia <10 PFU/ml) at all times tested. No virus was detected in any sera collected three days postchallenge.

**Fig 3:**
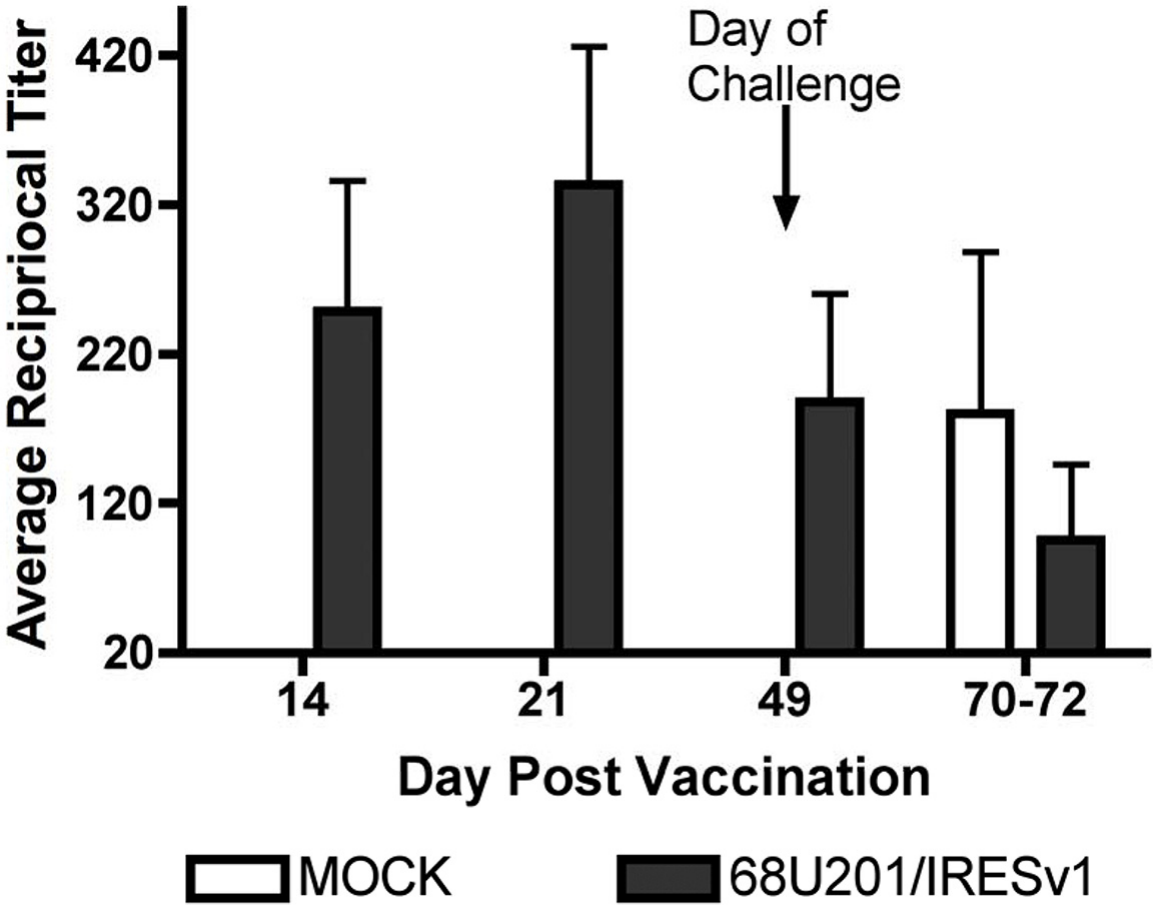
Neutralizing antibody titers in response to vaccination and challenge. Individual sera was tested for their ability to neutralize strain 68U201 VEEV at the indicated timepoints. Average PRNT_80_ values are shown. Values less than 20 are considered negative. Bars denote standard error.

The levels of anti-VEEV antibodies produced from vaccination and challenge were determined by PRNT. Each NHP was bled on days 14, 21 and 49 following vaccination to assess vaccine immunogenicity. Neutralizing antibodies were observed in all vaccinated individuals on day 14, and at all times tested thereafter (Fig 3). Only after challenge did unvaccinated NHP develop detectable antibody titers. Interestingly, an anamnestic response was not observed in all vaccinated NHPs after challenge.

Necropsies were performed on all NHP on days 21–23 post challenge to ensure that no residual VEEV was detectable in the tissues. Samples of lymph nodes (axillary, bronchial and inguinal), brain, kidney, liver, lung and spleen were homogenized and clarified supernatants were tested by cytopathic assay for the presence of infectious virus. No cytopathic effects were observed in any organ tested, indicating undetectable levels of persistent infectious virus in these subjects.

## Discussion

We previously described the safety and efficacy of the 68U201/IRESv1 vaccine in CD1 mice [11]. Here, these findings were expanded using a more relevant model for human infection, cynomolgus macaques. Unlike mice, which uniformly develop overt encephalitis and succumb to VEEV infection, NHP develop a non-fatal disease accompanied with fever that is typical of human disease [9, 10]. Using this superior model, the 68U201/IRESv1 vaccine provided complete protection against all measures of disease with a single dose. Vaccinated individuals all produced robust neutralizing antibody titers by day 14 post vaccination, which persisted until challenge at day 49.

Our data suggest that 68U201/IRESv1 is better at protecting against VEE than the current IND vaccine TC-83. Approximately 40% of human TC-83 recipients report some degree of febrile illness following vaccination [13]. Furthermore, TC-83 does not produce an antibody response in all individuals, and a booster of inactivated virus (C84) for non-responders [14]. Like humans recipients, not all cynomolgus macaques that receive the TC-83 vaccination seroconvert, and not all animals that develop neutralizing antibodies are protected against disease when with the IAB VEEV strain Trinidad donkey, from which TC-83 was derived [15]. These data highlight the need for a more efficacious vaccine to protect against aerosol exposure, particularly for those laboratory workers who may be at the highest risk for that route of infection as well as for biodefense purposes.

Several efforts have been made in to try to improve upon the efficacy and safety of TC-83 [16]. Two such vaccines were generated on the basis of the site-directed mutagenesis of furin cleavage sites in the VEEV genome: V3526 and IE1150K on the subtype IAB and IE backbones respectively [16]. Whereas 68U201/IRESv1 induced complete seroconversion and high neutralizing antibody titers against subtype IE VEEV, both V3526 and IE1150K induced incomplete seroconversion and lower neutralizing titers in cynomolgus macaques [17]. Additionally, V3526 demonstrated some signs of neurovirulence in the same nonhuman primate model [18]. Overall, 68U201/IRESv1 elicits higher neutralizing antibody titers and protects more effectively against clinical disease, as defined by an absence of fever and viremia, than any previously described, live-attenuated vaccine for VEE.

Protection with 68U201/IRESv1 was produced in the absence of a vaccine-induced viremia, which is a desirable characteristic for vaccines against arboviruses, especially if used in nonendemic locations. Indeed, this worst-case scenario was observed in nature during the VEEV epidemic in the 1970’s. TC-83 was isolated from mosquito pools collected in Louisiana, where horses were not vaccinated but where vaccination did occur in nearby Texas in an attempt to stop the spread of the epizootic/epidemic [19]. In addition to the lack of 68U201/IRESv1 viremia in vaccinated macaques and mice, the inability of this vaccine to infect mosquito cells due to inefficient EMCV IRES-mediated translation in insects provides a further safety advantage [11].

In summary, we have demonstrated the safety and efficacy of the 68U201/IRESv1 vaccine candidate in a human-relevant primate model. These results, together with those from our previous murine studies, underscore the promise of this vaccine for human use to prevent widespread VEE in the Americas and the risk of this biothreat agent.
