# Correction: IRES-Containing VEEV Vaccine Protects Cynomolgus Macaques from IE Venezuelan Equine Encephalitis Virus Aerosol Challenge

**DOI:** 10.1371/journal.pntd.0003912

**Published:** 2015-06-24

**Authors:** The PLOS Neglected Tropical Diseases Staff

## Notice of Republication

This article was republished on June 12, 2015, to correct an omission in the Editor section of the article. The Editor of this article was Ann M. Powers. The publisher apologizes for this error. Please download this article again to view the correct version.
